# Supplementary material for: High viral suppression rates among PLHIV on dolutegravir who had an initial episode of viral non-suppression in Uganda September 2020–July 2021
Source: PLoS One. 2024 Jun 26;19(6):e0305129. doi: 10.1371/journal.pone.0305129 (PMC11206940; doi:10.1371/journal.pone.0305129)
Supplement: Attachment — Submitted filename: Response to reviewer comments April 2024.docx [file pone.0305129.s003.docx]

**Department of Health and Human Services**

Centers for Disease Control and Prevention (CDC)

April 22^nd^, 2024

To the Editor,

We are re-submitting our revised manuscript, *High Viral Suppression Rates Among PLHIV on Dolutegravir who had an Initial Episode of Viral Non-Suppression in Uganda September 2020–July 2021*, for consideration for publication in *PLOS ONE*, following reviews received on March 12, 2024. We would like to thank the Editors and Reviewers for their comments on our original submission, which were helpful in improving the manuscript.

We have updated the manuscript addressing the comments of the Editors and Reviewers, using the track function in Word; we are also submitting a version of the file without any track changes, per the instructions. Below, please find responses to each of the reviewers’ comments (in italics).

We thank you for considering our paper for publication in *PLOS ONE*.

Sincerely,

**Grace Namayanja MBChB, MMed, MSc**

HIV Treatment Specialist

Division of Global HIV & TB | Center for Global Health

U.S. Centers for Disease Control and Prevention (CDC)-Uganda

Email: [uwo9@cdc.gov](mailto:uwo9@cdc.gov)

+256 772 139074

**“Response to reviewers**

**Comments to the Author**

1. If the authors have adequately addressed your comments raised in a previous round of review and you feel that this manuscript is now acceptable for publication, you may indicate that here to bypass the “Comments to the Author” section, enter your conflict of interest statement in the “Confidential to Editor” section, and submit your "Accept" recommendation.

Reviewer #1: All comments have been addressed

Reviewer #2: (No Response)

**Grace Namayanja on behalf of the coauthors (GN):** *We thank the reviewers for the continued insights toward strengthening the manuscript.*

2. Is the manuscript technically sound, and do the data support the conclusions?

The manuscript must describe a technically sound piece of scientific research with data that supports the conclusions. Experiments must have been conducted rigorously, with appropriate controls, replication, and sample sizes. The conclusions must be drawn appropriately based on the data presented.

Reviewer #1: (No Response)

Reviewer #2: Partly

**GN:** *We have further strengthened the sections on Methodology (e.g., sample size calculation) and Discussion (e.g., limitations) to provide further clarity on the scientific rigor of our investigation. We have ensured that our statisticians have reviewed the narrative and analyses, including the Results section, to ensure the manuscript is technically sound and that the interpretation of the data support conclusions. We are happy to address any specific questions Reviewers may have.*

3. Has the statistical analysis been performed appropriately and rigorously?

Reviewer #1: (No Response)

Reviewer #2: I Don't Know

**GN:** *All analyses were conducted by trained, experience statisticians that were dedicated to this project. Further, analyses underwent extensive clearance review, both in Uganda and through support from CDC headquarters in Atlanta. We are confident in how the analyses were performed. We are happy to address any specific concerns Reviewers may have.*

4. Have the authors made all data underlying the findings in their manuscript fully available?

The [PLOS Data policy](http://www.plosone.org/static/policies.action#sharing) requires authors to make all data underlying the findings described in their manuscript fully available without restriction, with rare exception (please refer to the Data Availability Statement in the manuscript PDF file). The data should be provided as part of the manuscript or its supporting information, or deposited to a public repository. For example, in addition to summary statistics, the data points behind means, medians and variance measures should be available. If there are restrictions on publicly sharing data—e.g. participant privacy or use of data from a third party—those must be specified.

Reviewer #1: (No Response)

Reviewer #2: No

GN: *We have submitted an Excel file, which contains de-identified data. Please let us know if any additional information would be needed, keeping in mind the need to maintain patient confidentiality.*

5. Is the manuscript presented in an intelligible fashion and written in standard English?

PLOS ONE does not copyedit accepted manuscripts, so the language in submitted articles must be clear, correct, and unambiguous. Any typographical or grammatical errors should be corrected at revision, so please note any specific errors here.

Reviewer #1: (No Response)

Reviewer #2: Yes

GN: *Many thanks.*

6. Review Comments to the Author

Please use the space provided to explain your answers to the questions above. You may also include additional comments for the author, including concerns about dual publication, research ethics, or publication ethics. (Please upload your review as an attachment if it exceeds 20,000 characters)

Reviewer #1: (No Response)

Reviewer #2: While the authors have addressed some of my prior comments, others have not yet been addressed and the text was not updated to include important information. It would also be helpful if the authors can explain what changes have been made to the tables.

***GN*:** *Thank you for your feedback. We want to ensure that all comments are adequately responded to and addressed in the text, as needed. Are there specific comments that were not addressed? No changes have been made to the tables; were there specific concerns that you could share?*

Please include information about the follow up interval for each individual on a per-individual basis, i.e. the amount of time between the initial episode of viral non-suppression and the first follow up visit. Was the duration of that interval associated with suppression (in either direction)? The authors replied that the visits were meant to be one month apart, but did not indicate specific information about the interval or evaluate whether the interval was associated with suppression. For example, if someone didn’t come back until 6 weeks later, were they more likely to be non-suppressed? Please also address this question in the manuscript itself, not just the response here.

***GN*:** *Thank you for this observation.* *This is certainly a limitation, as we are unable to conduct analysis of the time between follow-up intervals for each individual, given the limitations of the CPHL LIMS database. We have more explicitly highlighted this limitation.*

Why do the authors think that the reported adherence decreased, but viral suppression increased, at the first follow up?

The authors replied that the paper shows “when ART adherence improves, the PLHIV get virally suppressed” – while I agree with this, the analysis also shows that viral suppression can happen before adherence improves.
At repeat VL testing, 80.8% (421/521) achieved viral suppression
Overall, at initial VL testing, … 94.1% (480/510) had good self-reported adherence despite VNS
Among 493 PLHIV who had a documented adherence assessment at repeat testing, 91.9% (453/493) had good self-reported adherence
What would explain that? Please also address this question in the manuscript itself, not just the response here

***GN***: *Excellent observation! Our thought is that patients might initially be biased to self-report adherence that is better than their actual adherence, and then when they encounter more personalized, one-on-one IAC sessions following VNS, they will provide more accurate self-reported adherence. We have updated the text to more directly explain this observation, as well as more explicitly cite this as a limitation.*

The section on line 160 (now line 176) does not seem appropriate for the main text of a manuscript, based on the way it is phrased and the way it describes individual investigators and explains why they are not co-authors. I haven’t seen something like this in the main text of a manuscript before. I recommend removing it and putting this information in acknowledgements, funding, etc. The specific text is pasted below, including the word “Response:” which seems to me like a response to a review question, and not something that should be in the manuscript.

Response: “CDC Uganda with support from the associate director of science, strategic information branch chief, CDC Uganda staff and Ministry of health among others developed a broad protocol that provides for review and use of retrospective routinely collected de-identified data. This protocol received approval from the CDC Science and integrity branch and exemption from the Makerere School of Public Health to develop and write abstracts manuscripts from various data sources used in the country e.g., dashboards, databases, Registers. In our manuscript we utilized de-identified data from the Central Public Health laboratory (CPHL) database or Lab Information management system (LIMS). The CPHL conducts viral load testing on all samples national wide. This broad protocol had the senior leadership from the various institutions that CDC works with e.g., MOH – where Dr. Joshua Musinguzi is the Program Manager of the AIDS control program and CDC Uganda Strategic Information branch chief – Jenny Ward. Joshua and Jenny only served as the co-authors on the CDC broad protocol for programmatic data utilization. However, for this manuscript they were not specifically engaged in the work and therefore, we did not include them in as co-authors.

***GN*:** *We agree. And have now deleted it from the manuscript. Thank you!*

Line 282 indicates that repeat viral load testing took place if the patient reported >95% medication adherence. This would seem to strongly bias results towards finding viral suppression. Please include this earlier in the manuscript as a condition of the specific population under investigation (ie, those with good medication adherence), and include this as an overall limitation to the study.

***GN*:** *Thank you for the comment. The basic issue stems from two study limitations: first, the use of patient self-reported data on adherence, which might differ from actual adherence; and second, limitations in the database, for which we had to assume that the national guidelines were met to order the second viral load test (i.e., 3 IAC sessions with >95% self-reported adherence). We have more explicitly addressed this in the revised Discussion, especially in the limitations section. Thank you again!*

7. PLOS authors have the option to publish the peer review history of their article ([what does this mean?](https://journals.plos.org/plosone/s/editorial-and-peer-review-process#loc-peer-review-history)). If published, this will include your full peer review and any attached files.


If you choose “no”, your identity will remain anonymous but your review may still be made public.


**Do you want your identity to be public for this peer review?** For information about this choice, including consent withdrawal, please see our [Privacy Policy](https://www.plos.org/privacy-policy).

Reviewer #1: **Yes:** Akudo Ikpeazu MD, MPH, DrPH

Reviewer #2: No
